# Determinants of Non-Vaccination against Pandemic 2009 H1N1 Influenza in Pregnant Women: A Prospective Cohort Study

**DOI:** 10.1371/journal.pone.0020900

**Published:** 2011-06-14

**Authors:** Romain Freund, Camille Le Ray, Caroline Charlier, Carolyn Avenell, Van Truster, Jean-Marc Tréluyer, Dounia Skalli, Yves Ville, François Goffinet, Odile Launay, for the Inserm COFLUPREG Study Group

**Affiliations:** 1 Inserm U953, Recherche Epidémiologique en Santé Périnatale et Santé des Femmes et des Enfants, Université Pierre-et-Marie-Curie, Paris, France; 2 Université Paris Descartes, Faculté de médecine, Paris, France; 3 Assistance Publique-Hôpitaux de Paris (AP-HP), Hôpital Necker-Enfants Malades, Service des Maladies Infectieuses et Tropicales, Centre d'Infectiologie Necker-Pasteur, Paris, France; 4 Inserm CIC BT505, AP-HP, Hôpital Cochin, CIC de Vaccinologie Cochin Pasteur, Paris, France; 5 Assistance Publique-Hôpitaux de Paris, Hôpital Necker-Enfants Malades, Unité de Recherche Clinique, Paris, France; 6 Assistance Publique-Hôpitaux de Paris, Hôpital Saint Vincent de Paul, Maternité, Paris, France; 7 Assistance Publique-Hôpitaux de Paris, Hôpital Necker-Enfants Malades, Maternité, Paris, France; University of Texas Medical Branch, United States of America

## Abstract

**Background:**

In October 2009, the French government organized a national-wide, free of charge vaccination campaign against pandemic H1N1 influenza virus, especially targeting pregnant women, a high risk group for severe illness. The study objective was to evaluate pandemic flu vaccine uptake and factors associated with non-vaccination in a population of pregnant women.

**Methodology/Principal Findings:**

In a prospective cohort conducted in 3 maternity hospitals in Paris, 882 pregnant women were randomly included between October 12, 2009 and February 3, 2010, with the aim to study characteristics of pandemic influenza during pregnancy. At inclusion, socio-demographic, medical, obstetrical factors and those associated with a higher risk of flu exposition and disease-spreading were systematically collected. Pandemic flu vaccine uptake was checked until delivery. 555 (62.9%) women did not get vaccinated. Determinants associated with non-vaccination in a multivariate logistic regression were: geographic origin (Sub-Saharan African origin, adjusted Odd Ratio aOR = 5.4[2.3–12.7], North African origin, aOR = 2.5[1.3–4.7] and Asian origin, aOR = 2.1[1.7–2.6] compared to French and European origin) and socio-professional categories (farmers, craftsmen and tradesmen, aOR = 2.3[2.0–2.6], intermediate professionals, aOR = 1.3[1.0–1.6], employees and manual workers, aOR = 2.5[1.4–4.4] compared to managers and intellectual professionals). The probability of not receiving pandemic flu vaccine was lower among women vaccinated against seasonal flu in the previous 5 years (aOR = 0.6[0.4–0.8]) and among those who stopped smoking before or early during pregnancy (aOR = 0.6[0.4–0.8]). Number of children less than 18 years old living at home, work in contact with children or in healthcare area, or professional contact with the public, were not associated with a higher vaccine uptake.

**Conclusions/Significance:**

In this cohort of pregnant women, vaccine coverage against pandemic 2009 A/H1N1 flu was low, particularly in immigrant women and those having a low socio-economic status. To improve its effectiveness, future vaccination campaign for pregnant women should be more specifically tailored for these populations.

## Introduction

In June 2009, World Health Organization (WHO) raised the pandemic alert level to the highest level of 6. Although this pandemic was not at the scale expected by the public health services, it can be used as an example of a general mobilization of national health systems in a global campaign of vaccination [1]. For these reasons, data from the French 2009–2010 vaccination campaign can be used to improve the coverage and effectiveness of a future vaccination campaign in case of a new influenza pandemic.

According to numerous studies, pregnant women are considered to be at higher risk of severe illness from seasonal [2], [3] and pandemic influenza [4], [5], [6], [7]. Therefore, WHO [8], American Centers for Disease Control and Prevention of Disease (CDC) [9], European Centre for the Control and Prevention Diseases (ECDC) [10], European Commission, Health Security Committee (HSC)/ Early Warning and Response System (EWRS) [11], and the French Advisory Council for Public Health (HCSP) [12] define pregnancy as a high-risk priority group for vaccination. French authorities recommended pandemic H1N1 vaccination with a single dose of an adjuvanted-free vaccine (Panenza®) for all pregnant women after the first trimester [13].

The vaccination campaign began in France on November 9, 2009, according to an order of priority for people at risk of severe illness as predefined by the HCSP [12], [14]. The vaccination was administered, free of charge, in centers dedicated to pandemic vaccine. On November 20, 2009, Panenza® was available and pregnant women asked to get vaccinated. The objective of the French strategy was to cover 85% of the overall French population [15]. The outcome of the campaign showed that, on January 18, 2010, only 7.95% of the French population was vaccinated and only 22.7% of pregnant women [16].

These data raise the question of possible disparities in vaccine coverage among the French pregnant women. Although vaccination was available and free for all, some socio-demographic characteristics may have influenced women's decision toward vaccination. Moreover, some factors that would normally promote vaccination such as the working conditions (e.g in contact with the public, children or the medical community), obstetrical and medical characteristics, may also modulate women's awareness of vaccine's usefulness in the high-risk population of pregnant women.

The objective of this study was to evaluate pandemic flu vaccine uptake and to analyze the determinants related to the non-vaccination against the pandemic flu virus in a population of pregant women. The data of 882 pregnant women randomly included in a prospective cohort study conducted during the 2009 French vaccination campaign were used to address this question.

## Methods

### Participants

COFLUPREG 〈〈COhort on FLU during PREGnancy〉〉 is a prospective study conducted in three tertiary maternity centers in Paris, France, to determine the clinical expression, the biological characteristics, and the maternal-fetal impact of pandemic influenza A/H1N1 occurring during pregnancy. Between October 12, 2009 and February 3, 2010, 919 pregnant women were randomly drawn among pregnant women that were followed in these maternity hospitals, in order to include 45 women each day and to obtain a representative sample of pregnant women followed in these maternity hospitals. Women aged ≥18 years, speaking and understanding French were eligible to participate if they were pregnant between 12 and 35 weeks of gestation and followed in one of the three maternity participating to the study. Main exclusion criteria were a virologically documented H1N1 influenza during the last 6 months and vaccination against influenza A/H1N1 before inclusion. From the 919 pregnant women included in COFLUPREG study, 37 were excluded due to withdrawal of consent (n = 3), delivery before the date of vaccine availability (n = 8), and loss of follow up (i.e. women who gave birth in another hospital and have had less than 3 follow-up visits) (n = 26). Thus, data from 882 pregnant women were included to study determinants associated with non-vaccination against A/H1N1 influenza virus.

### Procedures

At inclusion in the study the following data were collected: socio-demographic characteristics (mother age, geographic origin, lifestyle (single or couple), socio-professional category), medical factors (co-morbidity associated with a high-risk of occurrence of severe form of flu, flu symptoms since the beginning of pregnancy, seasonal flu vaccination in the previous 5 years, smoking), obstetrical characteristics (gestational age, gestity, twin pregnancy, parity, significant obstetrical history and current pregnancy complication) and factors associated with a higher risk of viral exposition and disease-spreading (number of children under 18 years old at home, work in contact with children, healthcare workers and professional with consistent contact with the public).

Co-morbidity associated with a risk of occurrence of severe flu was defined by the presence of at least one of the following diseases: chronic lung disease (including asthma), severe cardiopathy, severe chronic nephropathy, severe neuropathy, severe myopathy, sickle-cell disease, diabetes mellitus, immunodeficiency, morbid obesity and alcoholism with chronic hepatopathy.

Significant obstetric history was defined as having at least one of the following events: late miscarriage (between 14^th^ and 21^th^+6 days weeks of gestation), preterm delivery (between 22^th^ and 36^th^+6 days weeks of gestation), and history of pre-eclampsia/gestational hypertension, intrauterine growth restriction, fetal malformation or fetal death. Current pregnancy complication was defined as having at least one of the following complications: placenta prævia, pyelonephritis, pre-eclampsia/gestational hypertension, gestational diabetes mellitus, suspicion of intrauterine growth restriction, fetal malformation, threatened preterm delivery and premature rupture of membranes (PROM).

The women were followed by doctors or midwifes with monthly visits until delivery. During each visit, information on the occurrence of vaccination against 2009 A/H1N1, of flu symptoms or documented A/H1N1 infection were prospectively collected.

### Ethics

Written informed consent was obtained from each woman before enrollment. The protocol was conducted in accordance with the Declaration of Helsinki and French law for biomedical research, and was approved by the “Ile-de-France 3” Ethics Committee (Paris, France), on October 2, 2009; n°09-12075.

### Statistical methods

For each variable, the choice of the reference class was made as in adequacy with literature. The reference class was the one known to have the highest vaccination rate. When the knowledge did not exist in literature, the reference was the class with the highest frequency.

Data management and statistical analysis were done using STATA for Windows (Version 10.0 College Station, Texas, USA). To compare numbers and percentages, we used Chi2 test or Fisher's exact test if n<5 and predicted n<5.

Associations between determinants and the non-vaccination against pandemic flu were analyzed using univariate analysis. Determinants with a p-value less than 0.20 on univariate analysis were included in the final logistic regression. Population characteristics differed between the three maternity hospitals. For this reason, a cluster model was used by adjusting the logistic regression model on the maternity center. This adjustment was achieved by including the estimated variances Huber / White / sandwich [17] into the logistic regression model. A systematic research of interaction between determinants with a p-value less than 0.20 on univariate analysis was performed.

## Results

### Study population

The demographic profiles and the clinical characteristics of the study population are described in Table 1. Median age was 32.7 years [min, max: 18.8, 49.1], 47.5% of the women were primiparous, 14.2% had at least one co-morbidity and 11% had at least one significant obstetric history. The median term of pregnancy was 37.7 weeks of gestation [min: 22.4; max: 40.3]. Of the 882 pregnant women, 555 (62.9%) did not get pandemic A/H1N1 vaccine.

**Table 1:** Characteristics of the study population and determinants associated with non-vaccination against pandemic 2009 A/H1N1 influenza: univariate analysis.

	Totaln = 882 (%)	Vaccinatedn = 327 (%)	Non Vaccinatedn = 555 (%)	p-value†
**Maternity hospital**				
Saint Vincent de Paul	233 (26.4)	108 (46.4)	125 (53.7)	
Port Royal	431 (48.9)	132 (30.6)	299 (69.4)	
Necker Brune	218 (24.7)	87 (39.9)	131 (60.1)	<0.01
**Inclusion month**				
October	215 (24.4)	92 (42.8)	123 (57.2)	
November	338 (38.3)	189 (55.9)	149 (44.1)	
December	215 (24.4)	39 (18.1)	176 (81.9)	
January	111 (12.6)	7 (6.3)	104 (93.7)	
February	3 (0.3)	0	3 (100)	<0.01††
**Age, years**				
18–24	40 (4.5)	7 (17.5)	33 (82.5)	
25–34	547 (62.0)	206 (37.7)	341(62.3)	
≥35	295 (33.5)	114 (38.6)	181 (61.4)	0.03
**Geographic origin**				
French, European	657 (74.5)	281 (42.8)	376 (57.2)	
Sub-Saharan African	49 (5.56)	6 (12.2)	43 (87.7)	
North African	89 (10.1)	17 (19.1)	72 (80.9)	
Asian and Other*	87 (9.9)	23 (26.4)	64 (73.6)	<0.01
**Lifestyle** *				
Single	60 (6.8)	10 (16.7)	50 (83.3)	
Couple	821 (93.2)	317 (38.6)	504 (61.4)	<0.01
**Socio-professional category** *				
Farmers/craftsmen and tradesmen	33 (3.8)	10 (30.3)	23 (69.7)	
Managers, intellectual professionals	371 (42.1)	168 (45.3)	203 (54.7)	
Intermediate professionals	209 (23.7)	80 (38.3)	129 (61.7)	
Employees and manual workers	158 (17.9)	44 (27.9)	114 (72.2)	
Unemployed people	110 (12.5)	25 (22.7)	85 (77.3)	<0.01
**Number of children under 18 years old at home**				
0	429 (48.6)	157 (36.6)	272 (63.4)	
1	314 (35.6)	125 (39.8)	189 (60.2)	
>1	139 (15.8)	45 (32.4)	94 (67.6)	0.31
**Job characteristic**				
Work in contact with the children				
- Yes	88 (10.0)	32 (36.4)	56 (63.6)	
- No	794 (90.0)	295 (37.2)	499 (62.9)	0.88
Healthcare worker				
- Yes	89 (10.1)	36 (40.5)	53 (59.6)	
- No	793 (89.9)	291 (36.7)	502 (63.3)	0.49
Professionals in contact with the public				
- Yes	403 (45.7)	146 (36.2)	257 (63.7)	
- No	479 (54.3)	181 (37.8)	298 (62.2)	0.63
**Seasonal vaccination in the previous 5 years** **				
Yes	99 (11.3)	47 (47.5)	52 (52.5)	
No	781 (88.8)	279 (35.7)	502 (64.3)	0.02
**Smoking** **				
No	671 (76.3)	243 (36.2)	428 (63.8)	
Stopping smoking before or early in pregnancy	115 (13.1)	52 (45.2)	63 (54.8)	
Yes**	94 (10.7)	32 (34.0)	62 (66.0)	0.15
- <10/d	71 (77.2)	24 (33.8)	47 (66.2)	
- 10–19/d	14 (15.2)	4 (28.6)	10 (71.4)	
- >19/d	7 (7.6)	2 (28.6)	5 (71.4)	0.90
**Gestational age at inclusion (gestational weeks)**				
<22	515 (58.4)	193 (37.5)	322 (62.5)	
[22–28]	186 (21.1)	68 (36.6)	118 (63.4)	
>28	181 (20.5)	66 (36.5)	115 (63.5)	0.96
**Gestity**				
1	288 (32.7)	112 (38.9)	176 (61.1)	
>1	594 (67.4)	215 (36.2)	379 (63.8)	0.44
**Twin pregnancy**				
Yes	39 (4.4)	21 (53.9)	18 (46.2)	
No	843 (95.6)	306 (36.3)	537 (63.7)	0.03
**Parity**				
0	419 (47.5)	155 (37.0)	264 (63.0)	
≥1	463 (52.5)	172 (37.2)	291 (62.9)	0.96
**At least one associated co-morbidity**				
Yes	125 (14.2)	39 (31.2)	86 (68.8)	
No	757 (85.8)	288 (38.0)	469 (62.0)	0.14
**Significant obstetrical history**				
Yes	97 (11.0)	27 (27.8)	70 (72.2)	
No	785 (89.0)	300 (38.2)	485 (61.8)	0.05
**Current pregnancy complication**				
Yes	32 (3.6)	14 (43.8)	18 (56.3)	
No	850 (96.4)	313 (36.8)	537 (63.2)	0.43
**Flu symptoms before the inclusion**				
Yes	80 (9.1)	38 (47.5)	42 (52.5)	
No	802 (90.9)	289 (36.0)	513 (64.0)	0.04

† Chi 2, p-value<0.20, included in the final logistic regression model.

†† Fischer exact Test.

*1 missing value.

**2 missing values.

### Factors associated with pandemic A/H1N1 vaccine uptake

#### Univariate analysis (Table 1)

Socio-demographic determinants significantly associated with the non-vaccination against 2009 A/H1N1 influenza virus were maternal age, geographic origin, lifestyle, and socio-professional categories.

Occurrence of a flu symptom since the beginning of the pregnancy was associated with a lack of 2009 A/H1N1 influenza vaccination (p = 0.04). On the opposite, no association was found between vaccine uptake and the presence of a co-morbidity associated with higher risk of severe viral infection (p = 0.14).

Obstetric factors, significantly associated with 2009 A/H1N1 influenza non-vaccination, were twin pregnancy and significant obstetric history. None of the current pregnancy complications was significantly associated with non-vaccination.

The lack of seasonal flu vaccination in the previous 5 years and smoking during pregnancy were correlated with A/H1N1 non-vaccination. None of the factors associated with a higher risk of exposition and disease-spreading to the virus (i.e. high number of children under 18 living at home or job characteristics) was associated with influenza A/H1N1 non-vaccination.

#### Multivariate analysis (Table 2)

**Table 2:** Determinants associated with non-vaccination against pandemic 2009 A/H1N1 influenza: multivariate cluster analysis including all determinants with a p-value<0.20 in the univariate analysis.

Variables	Odds-Ratios brut95%Confidence Interval	Adjusted OR95%CIWith Cluster
**Inclusion month**		
October, n = 215	**1.7 [1.2–2.4]**	**2 [1.7–2.3]**
November, n = 338	**1**	**1**
December, n = 215	**5.7 [3.7–8.9]**	**7.5 [6.9–8.2]**
January, n = 111	**18.8 [7.8–45.5]**	**35.4 [10.8–116]**
February, n = 3	**.**	**.**
**Age, years**		
18–24, n = 40	**2.8 [1.2–6.6]**	1.6 [0.3–9.1]
25–34, n = 547	1	1
≥35, n = 295	1.0 [0.7–1.3]	0.9 [0.7–1.2]
**Geographic origin**		
French, European, n = 657	**1**	**1**
Sub-Saharan African, n = 49	**5.4 [2.2–12.9]**	**5.4 [2.3–12.7]**
North African, n = 89	**3.2 [1.8–5.5]**	**2.5 [1.3–4.7]**
Asian and Other, n = 87	**2.1 [1.3–3.4]**	**2.1 [1.7–2.6]**
**Lifestyle** *		
Single, n = 60	**3.1 [1.6–6.3]**	2.2 [1.0–5.1]
Couple, n = 821	**1**	1
**Socio-professional category** *		
Farmers/craftsmen and tradesmen, n = 33	1.9 [0.9–4.1]	**2.3 [2.0–2.6]**
Managers, intellectual professionals, n = 371	1	**1**
Intermediate professionals, n = 209	1.3 [0.9–1.9]	**1.3 [1.0–1.6]**
Employees and manual workers, n = 158	**2.1 [1.4–3.2]**	**2.5 [1.4–4.4]**
Unemployed people, n = 110	**2.8 [1.7–4.6]**	2.3 [0.8–6.6]
**Seasonal vaccination in the previous 5 years** **		
Yes, n = 99	**0.6 [0.4–0.9]**	**0.6 [0.4–0.8]**
No, n = 781	**1**	**1**
**Smoking** **		
No, n = 671	1	**1**
Stopping smoking before or early in pregnancy, n = 115	0.7 [0.5–1.0]	**0.6 [0.4–0.8]**
Yes**, n = 94	1.1 [0.7–1.7]	1.2 [0.8–1.8]
**Twin pregnancy**		
Yes, n = 39	**0.5 [0.3–0.9]**	0.5 [0.2–1.2]
No, n = 843	**1**	1
**At least one associated co-morbidity**		
Yes, n = 125	1.4 [0.9–2.0]	1.2 [0.9–1.5]
No, n = 757	1	1
**Significant past obstetrical history**		
Yes, n = 97	**1.6 [1.0–2.6]**	1.7 [0.9–3.3]
No, n = 785	**1**	1
**Flu symptom before the inclusion**		
Yes, n = 80	**0.6 [0.4–1]**	0.7 [0.3–1.5]
No, n = 802	**1**	1

*1 missing value.

**2 missing values.

Factors associated with a lack of vaccination against pandemic flu were geographic origin (Sub-Saharan African origin, adjusted Odd Ratio (OR) 5.4, 95% CI [2.3–12.7], North African origin, adjusted OR 2.5, 95% CI [1.3–4.7] and Asian origin, adjusted OR 2.1, 95% CI [1.7–2.6] compared to French and European origin), socio-professional categories (farmers, craftsmen and tradesmen, adjusted OR 2.3, 95% CI [2.0–2.6], intermediate professionals, adjusted OR 1.3, 95% [1.0–1.6] and employees and manual workers, adjusted OR 2.5, 95% CI [1.4–4.4] compared to managers, intellectual professionals). The probability of not receiving A/H1N1 vaccine was lower among women vaccinated against seasonal flu in the previous 5 years (adjusted OR 0.6, 95% CI [0.4–0.8]) and among women who stopped smoking before or early during pregnancy (adjusted OR 0.6, 95% CI [0.4–0.8]) in comparison with non-smoking women (Table 2). The non–vaccination rate significantly increased after November (December, adjusted OR 7.5, 95% CI [6.9–8.2], January adjusted OR 35.4, 95% CI [10.8–116], compared to November).

## Discussion

Our study showed that, despite strong recommendations for vaccination against pandemic flu of pregnant women, a large proportion (62.9%) of pregnant women did not get the vaccine, particularly immigrant women and women having a low socio-economic status.

The percentage of non-vaccinated women is close to the estimation published by the French Institute for Public Health (InVS) reporting 77.3% of non-vaccinated pregnant women against pandemic flu [16]. The low vaccination coverage against influenza A/H1N1 in France and others countries could be partly explained by the controversy on the safety and efficacy of pandemic vaccines, and by a lack of knowledge about the risks of complications and mortality of influenza A/H1N1 [15], [18], [19]. In France, vaccination was performed in specifically dedicated centers located in non-medical public centers or gymnasiums, a fact that certainly reduced the convenience of the procedure and most of all the ability of family physicians to directly provide medical information promoting vaccination [20], [21], [22]. Indeed, in the United States, the percentage of pregnant women vaccinated was higher when vaccination was proposed by family physicians or healthcare professionals [18], [23]. However, other factors might have influenced pregnant women's decision regarding vaccination. For this reason, our study provides valuable complementary information about determinants of non-vaccination against the pandemic 2009 A/H1N1 influenza in pregnant women.

We found that foreign geographic origin was significantly associated with non-vaccination against pandemic flu. Previous studies have indeed shown a seasonal flu vaccine coverage disparity depending on geographic origin [24], [25]. This disparity can be explained by a lack of access to information among foreign populations or reticence about Occidental medicines. It has also been shown that a key determinant of vaccination access was the rate of vaccine reimbursement [26]. However since the vaccine was free and available for all in France, economical concerns should not have interfered with the choice of getting vaccinated. However, low incomes socio-professional categories did not get vaccinated as much as the other groups. This higher reticence towards vaccination in this group may reflect lower and biased access to medical information on vaccine benefits and safety.

Patients with medical or obstetrical co-morbidities are known to be a high risk group for severe pandemic flu. Thus, pregnant women with significant co-morbidity, pathological obstetric history or with significant disease during their current pregnancy should have been more vaccinated. However, they were not. This surprising trend has been evidenced elsewhere in another study focusing on seasonal influenza vaccine [27]. This failure might reflect a lack of awareness of healthcare professionals regarding the risks of A/H1N1 respiratory complications among pregnant women with medical or obstetrical co-morbidities and the necessity to encourage them to get vaccinated.

Furthermore, pregnant women at high risk of exposition and likewise disease-spreading should have been more vaccinated. However, women working with the public/ with children, and those with children living at home, were not more vaccinated than women at low risk of exposition and disease-spreading. This failure highlights the risk of large viral spreading beyond this group in the whole community. “More exposition, more risk to develop severe self-illness. More exposition, more risk to spread disease”: such strong messages should be more firmly diffused to the general population, including healthcare workers who did not get significantly more vaccinated than other working groups despite easier access to medical information. These surprising results were consistent with previous studies that have established this same lack of significant relationship between healthcare workers status and higher level of seasonal influenza vaccination [3], [21], [27]. It may results from misinformation/misunderstanding about the safety and efficacy of vaccines, which should be improved in case of future pandemic flu vaccination campaign.

In addition, pregnant women who had a seasonal flu vaccination in the previous 5 years got more vaccinated than those who did not had seasonal vaccination in the previous 5 years. Globally, patients that believed in the safety and efficacy of seasonal vaccination were more likely vaccinated against pandemic influenza. Other studies have observed similar trends among people vaccinated against seasonal influenza who were more prone to get vaccinated the following years [20], [27]. This factor reveals that once one gets vaccinated, he is less reluctant to get vaccinated again. Therefore, an effort on vaccination communication by the media for a year could have a positive impact on revaccination during the following years.

The non–vaccination rate of pregnant women significantly increased after November. On November 20^th^, 2009, when the vaccination campaign for pregnant women began, the fear of A/H1N1 Influenza complications was at its maximum. Misinformation induced a vaccination drop that could partially explain the increased non–vaccination rate in pregnant women after November. Furthermore, only non-vaccinated women could be included in the cohort, a bias which may explain the lower vaccine coverage for women included after November.

To our knowledge, only one Turkish survey that was conducted in only 314 pregnant women with a very low rate of vaccination (8.9%) studied few sociological, demographic and medical determinants to access pandemic influenza vaccination [22]. The only significant determinant associated with non-vaccination was the occupation: working pregnant women being more vaccinated than pregnant housewives.

Data from the COFLUPREG prospective cohort allow us to study numerous determinants associated with the vaccination against 2009 H1N1 influenza. A large number (882) of women were randomly included and followed-up throughout the pandemic. They were interviewed monthly regarding their vaccination status. This design and the quality of the data reinforce the reliability of the results.

We studied the determinants associated with the effective vaccination against A/H1N1 influenza and not only the intention to get vaccinated. Two French survey-based studies have assessed the determinants associated with the intention to get vaccinated in the general French population [18], [28]. However, the high discrepancy between the intention to get vaccinated before the pandemic start (61% of the French population in June 27, 2009) [15] and the effective vaccination rate (7.95% of the French population at the end of the pandemic) is a major limitation of these studies [16].

Our study has several limitations. First, the pregnant women sample comes from three university maternity hospitals in Paris. The results such as the vaccination incidence and the socio-demographic factors distribution cannot be extrapolated to all French pregnant women. But, this limitation does not interfere with the analysis of associations between studied determinants and non-vaccination among pregnant women. Secondly, women who accepted to participate to the COFLUPREG study were possibly influenced to get vaccinated. Yet, this influence seems to be low regarding the non-vaccination rate among pregnant women in our study (62.9%) which is similar to the French national estimation (77.3% by the InVS).

In conclusion, in a large prospective study conducted in pregnant women during the 2009 H1N1 influenza pandemic, the vaccination coverage against A/H1N1 influenza was low (62.9% of non-vaccinated women), particularly in immigrant women and those having a low socio-economic status. Our study provides unique data analyzing the reasons for the failure of a national vaccination campaign and yields trails for subsequent vaccination campaigns targeting high risk populations.
